# Role of community health outreach program “living for health”® in improving access to federally qualified health centers in Miami-dade county, Florida: a cross-sectional study

**DOI:** 10.1186/s12913-015-0826-z

**Published:** 2015-04-28

**Authors:** Aws Almufleh, Tori Gabriel, Laura Tokayer, Mary Comerford, Ahmed Alaqeel, Paul Kurlansky

**Affiliations:** Cardiac Sciences Department, King Saud University, Riyadh, Saudi Arabia; Internal Medicine Department, McGill University, 687 Pine Avenue West, Montreal, QC Canada; Education and Prevention Department, Florida Heart Research Institute, Miami, FL USA; Data Management Department, Florida Heart Research Institute, Miami, FL USA; Comerford Associates, Miami, FL USA; Division of Neurosurgery, Department of Surgery, King Saud University, Riyadh, Saudi Arabia; Department of Neurosurgery, University of Calgary, Alberta, Canada; Research Department, Florida Heart Research Institute, Miami, FL USA

**Keywords:** Health care quality, Access and evaluation, Community health centers, Vulnerable population

## Abstract

**Background:**

Care of the underserved remains one of the most compelling challenges to American healthcare. Federally Qualified Health Centers (FQHCs) address uninsurance and underinsurance by providing primary and preventive care to vulnerable populations with fees charged based on ability to pay. Our goal is to study the effectiveness of FQHCs system in engaging patients and the barriers to utilization, which have not been well defined.

**Methods:**

Retrospective analysis was performed on data from “Living for Health” (L4H) program participants from 2008 to 2012. Univariate and multivariate logistic regression analysis were performed to determine factors associated with FQHC utilization.

**Results:**

Among 9453 subjects screened, 1889 were referred to a FQHC, but only 201(11%) actually sought treatment. Public insurance, non-Hispanic ethnicity, and hypertension were associated with higher rates of FQHC utilization. Inability to afford costs, cultural factors and inflexible appointment times were the most common reasons for FQHC underutilization.

**Conclusion:**

The current status of FQHC utilization is sub-optimal. Community outreach programs like L4H can improve the access and utilization of FQHCs.

## Background

Cardiovascular disease remains the leading cause of death among both men and women in the United States affecting 17.6 millions of the population in 2010 and responsible for 787 650 (31.9%) of 2 468 435 deaths [1]. It is also responsible for significant morbidity, with an estimated 620 000 Americans having a new heart attack and about 295 000 having a recurrent attack every year. Essential to the prevention of cardiovascular morbidity and mortality is not only the identification of risk, but also the effective provision of healthcare to those with known risk factors [2]. To access this most needed healthcare, people who lack health insurance in the United States have the option of approaching safety net providers including Federally Qualified Health Centers (FQHCs) which are Community Health Clinics that have been approved for the main purpose of enhancing the provision of primary care and preventative services in underserved communities with charges for services on a sliding-scale basis. According to the Institute of Medicine definition, to qualify for FQHC, a center must maintain an “open door” offering access to patients regardless of their ability to pay and must dedicate a substantial portion of its patient share to be of uninsured, Medicaid and other vulnerable strata of the population [3]. Essentially, the FQHCs are funded by project grants and cost-based reimbursement for services provided under medicare and Medicaid [4]. In 2011, the number of FQHCs reached 1128 and served over 20 million patients, around 80% of who were uninsured and Medicaid beneficiaries.

Despite that, many patients who are in need of medical care still decline to utilize the FQHCs [5]. Indicative of this discrepancy is the fact that there are 48.6 million uninsured people in the US, but only 7.3 million actually paid a visit to the FQHCs [5]. Furthermore, in an analysis of National Health Institute Survey data 1997-2006, Hoffman and Schwartz reviewed over 76,000 non-elderly adults who were known to have at least one major chronic disease. They collected the data on participants’ insurance status and access of care, reporting 34.4% of uninsured had no usual source of medical care, 25.8% had no doctor visit in the last year and over 81% reported no specialist visit in the last year [6]. It is worth-mentioning that lack of primary care can result in increased emergency room use and increased health care costs [7,8].

The United States federal government recognizes the importance of promoting utilization of FQHC by the uninsured and underinsured which is exemplified by the provision in the Patient Protection and Affordable Care Act on improving access to FQHC [9,10]. Several studies have shown that community health centers including FQHCs save the health system millions of dollars partly by reducing unnecessary emergency visits of the uninsured and underinsured [7,11]. Therefore evaluating efficiency of FQHCs patients’ outreach and identifying barriers to their utilization can aid in optimizing access to care in vulnerable communities. This is broadly influenced by societal factors, health services-related factors and individual factors [12]. Certain barriers have been described in the literature including lack of health insurance [7], perceived complexities in healthcare system navigation and payer status, fear of deportation among undocumented residents, limited hours of FQHC operation, limited walk-in hours, and long wait times [8,13]. Lack of sufficient enabling services including transportation, translation, on-site child-care, and case management were also frequently mentioned reasons for lack of accessibility [8,13,14].

“Living for Health®”(L4H), a community health outreach program, organizes free cardiovascular risk factors screening events to adults (age ≥ 18) in zip codes in Miami-Dade County which were designated by the federal government as medically underserved areas. It identifies residents with cardiovascular risk factors and refers them to their Primary care provider or –if unavailable- FQHCs for treatment and follow up. The program focuses on cardiovascular risk factors in an attempt to address the leading cause of death in the United States, heart disease [1].

We introduce the “match rate”, defined as the proportion of patients who end up following up at FQHC after being referred to them from the community because of medical reason, as a measure for FQHC accessibility and utilization efficiency. The importance of using the match rate is to provide an objective quantitative measure for FQHC access and utilization. Our main goal in the current study is to evaluate the degree of FQHC utilization and identify areas of potential improvement to better serve the poor and underserved.

## Methods

Institutional Review Board approval was obtained from the Western Institutional Review Board with waiver of consent. Using a cross-sectional study design, data from “Living for Health” (L4H) program participants from 2008 to 2012 was reviewed. Living for health is operated by Florida Heart Research Institute (FHRI), which is an independent not-for-profit organization with the mission to stop heart disease through research, education and prevention. The program is funded by The Health Foundation of South Florida, the United Way of Miami-Dade County, Aetna Foundation, and the Florida Heart Research Foundation, delivers free-of-charge cardiovascular risk factor screening events which seek to identify residents with undiagnosed or untreated high blood pressure, hyperlipidemia, diabetes and/or obesity in zip codes in Miami-Dade County which were designated by the federal government as medically underserved areas. The cardiovascular screening events take place in areas of common gathering within the identified zip codes with utilization of local media and cooperation with community leaders to advertise those events. Participants who are found to have clinical values outside the normal range are referred to either their personal physician or, if they do not have one, to one of the participating FQHCs in the area. L4H educators who have received training on recognizing abnormal health parameters decide on whether a participant needs referral and they make that decision. Physicians and community nurses provide this training to educators prior to starting the program. Along with the FQHC referrals-if indicated-, the participants at risk receive an intensive health education that is focused on proper diet, encouraging exercise, regular adherence to medications and follow up – if applicable- and avoiding risky behaviors (smoking, alcoholism, drugs. etc.). Furthermore, when referral – either to Family doctor or FQHC - is indicated, the patient is educated about available services, importance of approaching them and the process of enrollment and registration. After each free screening event, FHRI supplies the appropriate FQHC with a referral log (Name of participant, date of birth, address, daytime phone number, alternate phone, best time to call, what risk factor were they sent to follow-Glucose, blood pressure or cholesterol) The choice of which FQHC to refer the patient to depends on the location of event, address of the patient, existing agreements between certain FQHCs and FHRI, and most importantly patient’s choice. L4H team maintains follow-up with FQHCs up to 2 years post referral to determine if the participant referred does seek treatment at FQHC, i.e. matches or not.

### Subjects and settings

The study population consisted of L4H participants who fulfilled the following inclusion criteria; (1) adults (age ≥18 years) who consented to participate in one or more L4H screening events, (2) Participants with an abnormal clinical or laboratory value in blood glucose, cholesterol or blood pressure specifically systolic blood pressure > 140 mmHg, diastolic blood pressure > 90 mmHg, total cholesterol level > 240 mg/dL, TC/HDL ratio ≥ 4.1 or glucose level > 200 mg/dL thereby prompting referral for further follow-up (3) participants who require referral but do not have a Primary Care Provider and consented to be referred to FQHCs.

### Data collection

Data on demographics, insurance state, past medical and family histories were collected through bilingual (English-Spanish) self-administered questionnaires that were filled by participants at the beginning of each screening event. FHRI employees measured height, weight, BMI, finger stick for glucose and lipid profile for each participant and entered that into the database soon afterwards. The involved FQHCs had an agreement with FHRI for sharing information regarding patients’ matching; that data was used to calculate the match rate. Participants who consented to sharing their information with FQHC did so by signing a form during the screening events. The database was stored at the FHRI office with only relevant and clinically important data being shared with concerned FQHC.

As there was (1688) referred to FQHCs but did not match compared to patients who were matched (201), we conducted a follow-up phone survey 3-6 months on average after the screening event to further understand the reasons for not matching.

### Study variables

Demographics, insurance status, general health status self-reported ratings, family history of heart disease, and past medical histories of diabetes, hypertension, heart disease, and hypercholesterolemia. Independent variables also included height, weight, body mass index (BMI), blood pressure, non-fasting blood glucose and lipid profile values were included in the study. Data analysis of each variable was performed for all participants who completed that certain variable; missing data were excluded with pairwise deletion method.

### Data analysis

The Statistical package for the Social Sciences (SPSS) 16 was used for data analyses. Univariate analyses were performed to test the relationship between demographic, historical and clinically measured variables, and matching as a dichotomous outcome variable. Chi-square test was applied to the following variables: age, gender, race and ethnicity, insurance status, participant’s self-reported health rating, and self-reported history of diabetes, hypertension, hyperlipidemia or heart disease and measured hypertension, elevated cholesterol, and elevated glucose. Univariate and multivariate logistic regression analysis were performed. P < .05 was considered the cutoff value for significance.

## Results

**Figure 1:**
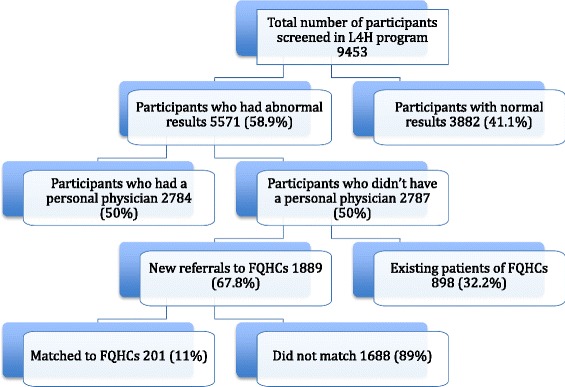
Flow chart of L4H program participants and the proportion referred and matched to FQHC.

Out of the 9453 people screened in L4H program, 5571 (58.9%) were found to have abnormal clinical values thereby warranting physician referral. Two thousands, seven hundreds and eighty-seven (50% of participants with abnormal clinical values) of those were found not to have a personal physician and were therefore referred to a FQHC. Eight hundred ninety eight (32.2%) of those participants were existing patients of an FQHC; the remaining 1889 (67.8%) patients were new referrals of whom only 201 (11%) ended up matching to the FQHCs (Figure 1).

Baseline demographics, insurance status, self-reported health status, and last physical exam done were compared between participants who matched and those who did not match (Please see Table 1). For the participants’ age and gender, there was no significant difference between the two groups. Insurance status, however, was significantly different between the two groups; compared to the public insurance, those who were not insured were 56% less likely to match to FQHC (OR .44, 95% CI [.24-.80]). Insurance status was also significantly associated with how recent a patient has visited a doctor (P < 0.001); Forty-one percent of uninsured individuals in our study had the last doctor’s visit 2 or more years prior to our screening event as compared to 11% in publicly insured and 16% in privately insured participants.

**Table 1:** **Bivariate analysis of the relationship between baseline demographics, last physical exam, and insurance characteristic and matching**

**Demographic variable**	**Distribution**	**Matched (%)**	**Unmatched (%)**	**Odds ratio of matching**	**95% CI**
**Ordinal variable:**	18- < 25 years	8	106	0.743	(0.30-1.86)
-Age	25- < 65 years	180	1441	1.23	(0.68-2.22)
	> = 65 years	13	128	1.00	Reference
	Total	N = 201	N = 1675		
-Self-reported health status	Excellent or very good health	8	88	0.63	(0.26-1.54)
	Good	90	187	1.11	(0.58-2.13)
	Fair or poor	16	111	1.00	Reference
	Total	N = 54	N = 386		
-Last time you had a physical exam	Within the last year	53	438	0.98	(0.70-1.37)
	More than 1 year ago	143	1155		
	Total	N = 196	N = 1593		
**Nominal variables:**	Female	123	952	1.213	(0.90-1.64)
-Gender	Male	78	732		
	Total	N = 201	N = 1684		
-Insurance status	Public Insurance (Medicaid or Medicare)	14	53	2.30	(1.25-4.23)
	Private Insurance	5	48	0.91	(0.36-2.31)
	No insurance	179	1559	1.00	Reference
	Total	N = 198	N = 1660		
-Race and ethnicity	Non-Hispanic Black	61	368	1.63	(1.17-2.27)
	Non-Hispanic White	12	85	1.39	(0.74-2.62)
	Hispanic	120	1182	1.00	Reference
	Total	N = 193	N = 1635		

Race and ethnicity, as well, significantly differed between the two groups. Non-Hispanic Blacks were 63% more likely to match than Hispanics (OR 1.63, 95% CI [1.17-2.27]). Participants self-reported health status and reported prior physical exam date were not significantly associated with matching.

Similarly, self-reported chronic diseases and measured values of clinical parameters were compared between matched and unmatched groups (please see Table 2). Having diabetes, heart disease, and hypercholesterolemia were not significantly associated with matching status. However, having hypertension was significantly associated with matching, with those who have hypertension being 40% more likely to match (OR 1.40, 95% CI [1.02-1.92]). On the other hand, measured body mass index (BMI), random glucose, high-density lipoprotein (HDL), and total cholesterol/high density lipoprotein (TC/HDL) ratio were not significantly associated with matching. Total cholesterol was significantly associated with matching; those who had normal cholesterol were 41% more likely to match than those with high cholesterol (OR 1.41, 95% CI [1.05-1.90]).

**Table 2:** **Bivariate analysis of the relationship between self-reported chronic diseases and measured clinical parameters and matching**

**Variable**	**Distribution**	**Matched (%)**	**Unmatched (%)**	**Odds ratio of matching**	**95% CI**
**Nominal variable:**	Hypertensive	68	460	1.40	(1.02-1.92)
-Self-reported HTN	Non-hypertensive	122	1154		
	Total	N = 190	N = 1614		
-Self-reported Diabetes Mellitus	Diabetic	28	179	1.39	(0.90-2.13)
	Non-diabetic	161	1427		
	Total	N = 189	N = 1606		
-Self-reported Hyperlipidemia	Hyperlipidemic	69	497	1.25	(0.92-1.71)
	Non-hyperlipidemic	124	1117		
	Total	N = 193	N = 1614		
-Self-reported heart disease	Has heart disease	6	49	1.18	(0.50-2.79)
	Doesn’t have heart disease	145	1391		
	Total	N = 151	N = 1440		
**-Ordinal variables:**	Glu < 200 mg/dl	185	1591	1.48	(0.85-2.57)
-Measured high random blood glucose	Glu ≥ 200 mg/dl	16	93		
	Total	N = 201	N = 1684		
-Measured total Cholesterol	TC ≤ 240 mg/dl	118	846	1.41	(1.05-1.90)
	TC > 240 mg/dl	83	840		
	Total	N = 201	N = 1686		
-Measured High Density Lipoprotein	HDL ≤ 40 mg/dl	120	1015	0.98	(0.72-1.31)
	HDL > 40 mg/dl	81	668		
	Total	N = 201	N = 1683		
-Calculated TC/HDL ratio	TC/HDL < 4	103	780	1.22	(0.91-1.93)
	TC/HDL ≥ 4	98	903		
	Total	N = 201	N = 1683		
-Measured height and weight, BMI’	BMI ≤ 25	37	390	0.75	(0.52-1.09)
	BMI > 25	163	1287		
	Total	N = 200	N = 1677		

After fitting all variables that were significant in the univariate analysis in a logistic regression model (see Table 3), the adjusted OR for non-Hispanic Blacks became 2.19 (95% CI [(1.51-3.17)]) and that of self-reported hypertension (HTN) became 1.45 (95% CI [1.03-2.04]). After adjusting for all of the previous variables, absence of insurance and normal measured cholesterol were no longer significantly associated with the matching status. Participants, who were newly referred to a FQHC but did not match, were asked the question: “What is the primary reason why you did not see a physician?” (See Table 4). The majority 58.1% reported the reason to be either absence of insurance or inability to afford the expenses. 11.7% reports appointment delay from the FQHC as the primary reason; 13.4% complained of not having enough time to seek medical help.

**Table 3:** **Multivariate logistic regression analysis of the relationship between demographics, self-reported illnesses and measured health risks and matching**

**Variable**		**Adjusted odds ratio**	**95% confidence interval**
Insurance status	Public Insurance (Medicaid or Medicare)	1.68	(0.59-4.80)
	Private Insurance	0.53	(0.27-1.01)
	No insurance	1.00	Ref.
Race and ethnicity	Non-Hispanic Black	2.19	(1.51-3.17)
	Non-Hispanic White	1.33	(0.69-2.56)
	Hispanic	1.00	Ref
Self reported Hypertension (HTNsive/Non-HTNsive)		1.45	(1.03-2.04)
Measured total Cholesterol ( TC ≤ 200 mg/dl/TC > 200 mg/dl )		1.27	(0.92-1.76)

**Table 4:** **Unmatched participants’ answer to the question “What is the primary reason why you did not see a physician?”**

**Question**	**Distribution**	**Frequency**	**Percentage**
What is the primary reason why you did not see a physician?	1- No insurance	490	50.8%
	2- Can’t afford to go	73	7.3%
	3- Appointment delay	109	11.3%
	4- Do not feel I need to see a doctor	114	11.7%
	5- Not told I need to see a physician	1	0.1%
	6- Do not have time/have to work	130	13.4%
	7- Clinic did not call for appt	25	2.1%
	8- Other	18	3.2%
Describe the reason why you didn’t go to the FQHC?	The FQHC were not flexible in arranging the appointment	1	0.1%
	The FQHC required Tax return and other documents the patient could not provide	1	0.1%
	Will visit another physician in native country	1	0.1%
	Patient preferred to wait until they became Medicaid eligible	1	0.1%

## Discussion

To the best of our knowledge, this study represents the first reported investigation of factors influencing matching patients in need of medical care to FQHCs. Our study revealed that a large percentage (59%) of participants screened in L4H program had at least one abnormal clinical parameter warranting referral to a physician. This considerable proportion that required care for their newly diagnosed or pre-existing chronic diseases emphasizes the importance of programs like L4H in identifying and guiding patients to navigate their options of care in the health system. Furthermore, the fact that 67% of those who did not have a personal physician had never been to a FQHC before further highlights the importance of outreach programs in connecting patients without the ability to pay to FQHCs. The most common reason for referral was due to abnormalities in clinical values in patients who were otherwise asymptomatic. Without community screening, those patients may not seek medical care and their diseases would progress to a more complicated stage.

Lack of insurance, by itself, has been related to worse overall health outcomes including higher risk of premature death, mainly because of lack of reliable access to care [15-17]. Our finding that uninsured participants are significantly less likely to have had doctor’s appointment in the 2 years preceding the screening event further enforces that concept.

Despite the efforts of L4H screeners to connect patients who had no primary care physician to the FQHCs and despite FHRI’s efforts to assist clinics with recruitment by providing a referral log and educating the patients about the importance of having steady medical follow-up, only 11% were ultimately connected or “matched” to one of these centers. Although no similar study has previously reported this parameter, a match rate of 11% is disturbing, especially considering that Miami-Dade County has one of the most sophisticated FQHC networks in the country [9]. Therefore, discovering the barriers that have prevented most of the participants from pursuing medical care in the clinics is paramount in solving this major public health issue.

Participants who had no insurance were less likely to match among our study subjects. When participants who did not match were asked for the primary reason for not seeking care, 50.8% report the reason to be lack of insurance. Although the participants were told that FQHCs utilize a sliding fee scale in their provision of services, the inability to afford the clinic’s fees was the second most common reason given for not seeking care. This indicates that even the possibility of paying a small fee for health services was enough to discourage participants from approaching FQHCs.

Of note, the hypertensive patients in our study had 1.5 higher likelihood of matching, even after adjusting for other factors. This subgroup represents a slightly sicker participant group many of whom already had unfilled prescriptions for antihypertensive medication and therefore may have been more aware of their elevated risk for death or disability. It’s also notable that even when insurance status, hypertension, and other factors were adjusted, Hispanics, who constitute 65% of the population in Miami-Dade County were less likely to match than Black or White non-Hispanics [18]. Our study results alone cannot explain if this finding is confounded by other factors that we did not study (e.g. socioeconomic status, unemployment rate, etc..) or is a genuine indication of ethnic disparity.

Furthermore, our study showed lack of flexibility in FQHC appointments, appointments delay, and lack of appointments on afterhours to be among the reasons for low FQHCs utilization. The literature quotes other similar reasons including fear of reprisal due to illegal immigration to be correlated with underutilization of free community clinics [13]. Other frequently mentioned reasons include lower satisfaction with FQHC [19], perceived complexities in healthcare system navigation and payer status, limited hours of FQHC operation, long waiting times, and lack of sufficient enabling services including transportation, translation, on-site child-care, and case management [8,13,14].

While our study is one of the first to describe the factors associated with FQHC utilization, it still has it is own limitations. It has a cross sectional study design. Furthermore, FQHC system is heterogeneous across the United States, our study covered only one county and results may not be safely extrapolated to other counties. Finally, lack of long-term follow-up precluded linking matching to FQHCs to better control of chronic illnesses and improved survival.

Based on our results, we recommend that FQHCs work on improving the clinics’ ability to enroll new patients into benefit programs, expanding working hours, providing transportation, and childcare along with other enabling services. We also recommend them to support community outreach programs like L4H to identify at-risk patients and direct them to their best source of care. We believe that this will eventually result in more healthcare cost saving and will produce a healthier, more productive population.

## Conclusion

The current FQHCs’ access and utilization by uninsured and underinsured individuals is sub-optimal. It is hoped that community outreach screening programs like L4H can contribute to the solution by providing wellness visits (health screening and risk factors modification education) and encouraging many uninsured patients to seek care in FQHCs. More studies are needed to assess the FQHCs utilization in multiple counties over the United States and link that to long-term health outcomes.

## Abbreviations

FQHCs: Federally qualified health centers
L4H: Living for health
FHRI: Florida heart research institutex
SPSS: Statistical package for the Social Sciences
BMI: Body mass index
TC/HDL: Total cholesterol/high density lipoprotein
HTN: Hypertension
HDL: High-density lipoprotein
